# Online catalogue of the Coleção de Flebotomíneos (FIOCRUZ/COLFLEB), a biological collection of American sand flies (Diptera: Psychodidae, Phlebotominae) held at Fiocruz Minas, Brazil

**DOI:** 10.46471/gigabyte.52

**Published:** 2022-05-23

**Authors:** José Dilermando Andrade-Filho, Alanna Silva Reis, Carolina Cunha Monteiro, Paloma Helena Fernandes Shimabukuro

**Affiliations:** ^1^ Coleção de Flebotomíneos (FIOCRUZ/COLFLEB), Instituto René Rachou, Fiocruz Minas Avenida Augusto de Lima, 1715 - Barro Preto, 30190009, Belo Horizonte, Brazil; ^2^ Grupo de Estudos em Leishmanioses, Instituto René Rachou, Fiocruz Minas Avenida Augusto de Lima, 1715 - Barro Preto, 30190009, Belo Horizonte, Brazil

## Abstract

The Coleção de Flebotomíneos (“Phlebotomine Collection”; FIOCRUZ/COLFLEB) held at Fiocruz Minas is a curated biological collection comprising approximately 80 000 individual specimens of 370 species of sand flies (Diptera: Psychodidae, Phlebotominae). These were mostly collected from the Americas in the last 80 years by entomologists interested in understanding and controlling the vector-borne disease leishmaniases. Since 2010, the metadata of each biological specimen held in FIOCRUZ/COLFLEB, including the back catalogue of those deposited in previous decades, has been digitized. Here, we present the resulting electronic catalog containing records for 72,624 of the specimens, including all of the available provenance information associated with each specimen. The catalog is published online through the speciesLink network and the Sistema de Informação sobre a Biodiversidade Brasileira (SiBBr).

## Data description

### Background

Biological collections are repositories of biodiversity housing specimens and their related information, which can be used in various areas of scientific research [1]. These collections are especially important for insects of medical and veterinary importance, where accurate taxonomic identification is necessary for understanding and intervening in the epidemiology of vector-borne diseases [2]. The “Coleção de Flebotomíneos” (“Phlebotomine Collection”) is held at the Instituto René Rachou, Fiocruz Minas (FIOCRUZ/COLFLEB), a federal public health research institution in Belo Horizonte, Minas Gerais, Brazil. FIOCRUZ/COLFLEB is a biological collection of preserved sand flies (Diptera: Psychodidae, Phlebotominae). These small insects are of considerable medical and public health importance because their blood-feeding adult females transmit protozoan parasites of the genus *Leishmania*, the etiological agent of leishmaniases, in addition to other bacterial and viral pathogens, which infect both human and non-human vertebrates [3]. The insect specimens deposited in FIOCRUZ/COLFLEB come from many different research projects carried out over the last 80 years. These projects were undertaken in many different areas of leishmaniases transmission, as well as from wild environments where leishmaniasis has never been recorded. The deposited sand flies were usually collected using light traps and have been identified by highly trained specialists using available taxonomic keys [2, 4]. The specimens can support research in the areas of taxonomy and systematics [5–7], and their associated data can be used in ecological niche/species distribution modelling [8], among other applications [9].

**Figure 1.**
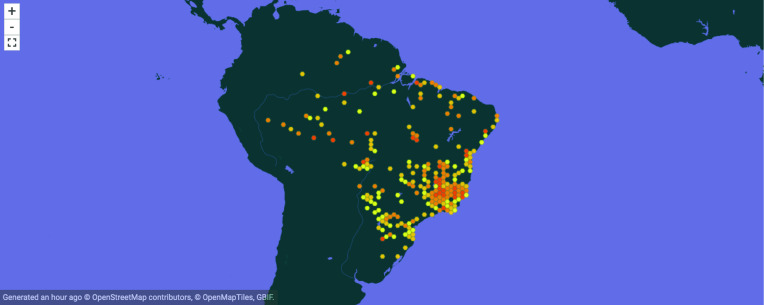
Interactive map of the georeferenced occurrences hosted by GBIF [12]. https://www.gbif.org/dataset/2a629a9a-38d1-496b-afbf-b4ff3b8fae60

The dataset reported here is the metadata for each individual sand fly specimen deposited in FIOCRUZ/COLFLEB since 1953. Our dataset has 57 fields, which for each individual sand fly specimen describes its (i) taxonomy (kingdom, phylum, class, order, family, genus, specificEpithet, infraspecificEpithet, scientificName, scientificNameAuthorship, taxonRank, vernacularName, typestatus); (ii) collection details, including the collectors (recordedBy), collection date, trapping method, trap identification number, collection site description (occurrenceRemarks, eventDate, eventTime, habitat, samplingProtocol, samplingEffort, eventRemarks); (iii) geolocation data (country, countryCode, stateProvince, county, Island, waterbody, locality, locationRemarks, decimalLongitude, decimalLatitude, georeferenceRemarks); and (iv) catalogue reference data (occurrenceID, catalogNumber, OtherCatalogNumbers). The associated data for each physical object in our collection has a paper card with many fields referring to specimen provider, location, quantity of specimens, and so on. Data from these cards have been digitized by a dedicated member of the collection staff since 2010. The data are provided in the Darwin Core format [10]. Our data are available in the Sistema de Informação sobre a Biodiversidade Brasileira (SiBBr), an online platform that integrates data and information about biodiversity and ecosystems. SiBBr is the Brazilian node of the Global Biodiversity Information Facility (GBIF) [11], an internationally recognized resource for collating biological occurrence data. Our dataset has been submitted to GBIF, and is publicly available for use by others there (see Figure 1) [12].

### Context

Phlebotomine sand flies are insects of medical importance because they are involved in the transmission of pathogens between human and non-human animals. Approximately 1,000 sand flies species have been described, of which 530 are known to occur in the neotropical and nearctic regions [13].

FIOCRUZ/COLFLEB was officially started in 1953 as part of the work of Professor Amilcar Vianna Martins and Alda Lima Falcão, in collaboration with the technician João Evangelista da Silva [14]. The number of specimens in the collection increased considerably during the 1960s and 1970s. In the last 20 years, fossil specimens, as well as voucher specimens from DNA-based barcoding studies and other epidemiological studies involving fieldwork, have continued to be deposited in the collection [7, 15].

FIOCRUZ/COLFLEB contains 922 type specimens belonging to 151 species, including holotypes, allotypes, paratypes, plesiotypes, cotypes, topotypes, homeotypes, syntypes and neotypes. FIOCRUZ/COLFLEB also has a diverse collection of fossil species of neotropical sand flies, currently comprising 47 ambers from the Dominican Republic, within which 162 sand flies of nine species are preserved. Additionally, FIOCRUZ/COLFLEB has over 700 voucher specimens deposited from continuing DNA barcoding studies.

Efforts to digitize the metadata of biological specimens held in FIOCRUZ/COFLEB have been ongoing since 2010. Among the biological collections of the various regional institutions that comprise Fiocruz [16], FIOCRUZ/COLFLEB has the most published online data. Our online catalog is also integrated into the speciesLink network [17] and the Sistema de Informação sobre a Biodiversidade Brasileira (SiBBr) [18].

## Methods

The sand flies held in FIOCRUZ/COLFLEB are adults and were collected using various methods, including resting collection from artificial and natural surfaces using either mechanical or manual aspirators (“pooters”); human-landing capture unbaited and CO_2_-baited CDC-like light traps; and Shannon traps. Collections were made in different environments, including domestic and peridomestic (i.e., houses and their surroundings in urban, suburban and rural areas) and relatively undisturbed sylvatic and wild area (e.g., remote forest areas). Our specimens come from all 27 states of Brazil, and 19 other different countries of the Americas.

The live-caught insects are permanently mounted on glass microscope slides and preserved in either Berlese medium or Canada Balsam, while the fossil specimens are kept in plastic vials. All types are labelled with color-coded labels.

## Data validation and quality control

Insects were identified by experienced taxonomists using keys available in the literature: those of Galati [2] and Young and Duncan [4].

The dataset is in Darwin Core format [10], with 57 possible terms available. All mandatory fields are present and have undergone screening in the FIOCRUZ IPT (Integrated Publishing Toolkit) [18], which is the GBIF software to provide data through their network. Metadata fields are also available on the online pages.

## Re-use potential

The data associated with the biological specimens deposited in FIOCRUZ/COLFLEB are of importance because they (i) describe the distribution of sand flies in different parts of Brazil, and (ii) have a good temporal coverage – some specimens date back to 1939, while the most recent deposit was recorded in 2021. These data can be used for many different applications, in different research areas, including taxonomy and systematics [5–7], and ecological niche modelling [8], among others [9], to vector control activities [19, 20].

## Data Availability

The data supporting this article are published through the FIOCRUZ – Oswaldo Cruz Foundation IPT [18] and are available under a CC0 waiver from GBIF [12].

## Editor’s note

This paper is part of a series of Data Release articles working with GBIF and supported by TDR, the Special Programme for Research and Training in Tropical Diseases, hosted at the World Health Organization [21].

## List of Abbreviations

FIOCRUZ/COLFLEB: Coleção de Flebotomíneos, Fiocruz Minas (“Phlebotomine Collection of Fiocriz Minas”); GBIF: Global Biodiversity Information Facility; IPT: Information Publishing Tool; SiBBr: Sistema de Informação sobre a Biodiversidade Brasileira (“Information System for Brazilian Biodiversity”); TDR: the Special Programme for Research and Training in Tropical Diseases.

## References

[1] Kamenski PA, Sazonov AE, Fedyanin AA Biological Collections: Chasing the Ideal. Acta Naturae, 2016; 8(2): 6–9. PMC4947984 27437135

[2] Galati EAB. Classification of Phlebotominae. In: Rangel EF, Lainson R (eds), Flebotomíneos do Brazil. Rio de Janeiro: Editora Fiocruz, 2003; pp. 23–175.

[3] Rangel EF, Lainson R. Flebotomíneos do Brasil. In: Rangel EF, Lainson R (eds), Flebotomíneos do Brazil. Rio de Janeiro: Editora Fiocruz, 2003.

[4] Young DG, Duncan MA. Guide to the identification and geographic distribution of Lutzomyia sandflies in Mexico, The West Indies, Central and South America (Diptera: Psychodidae). Mem. Am. Entomol. Inst., 1994; 54: 1–881.

[5] Costa GDS, Júnior AMP, Arley Costa Pessoa F New Records of Phlebotomine Sand Flies (Diptera: Psychodidae) From the Western Brazilian Amazon and the Description of the Female of Pintomyia fiocruzi. J. Med. Entomol., 2020; 57(4): 1328–1333. doi:10.1093/jme/tjaa030. 32099998

[6] Sábio PB, Brilhante AF, Quintana MG On the Synonyms of *Psathyromyia (Psathyromyia) shannoni* (Dyar, 1929) and *Pa. bigeniculata* (Floch & Abonnenc, 1941) and the Resuscitation of *Pa. pifanoi* (Ortiz, 1972) With the Description of Its Female (Diptera: Psychodidae: Phlebotominae). J. Med. Entomol., 2016; 53(5): 1140–1147. doi:10.1093/jme/tjw094. 27358041

[7] Rodrigues BL, Baton LA, Shimabukuro PHF. Single-locus DNA barcoding and species delimitation of the sandfly subgenus Evandromyia (Aldamyia). Med. Vet. Entomol., 2020; 34(4): 420–431. doi:10.1111/mve.12458. 32590879

[8] Andrade-Filho JD, Scholte RGC, Amaral ALG Occurrence and Probability Maps of *Lutzomyia longipalpis* and *Lutzomyia cruzi* (Diptera: Psychodidae: Phlebotominae) in Brazil. J. Med. Entomol., 2017; 54(5): 1430–1434. doi:10.1093/jme/tjx094. 28472338

[9] Rodrigues BL, Costa GDS, Shimabukuro PHF. Identification of Bloodmeals from Sand Flies (Diptera: Psychodidae) Collected in the Parque Nacional do Viruá, State of Roraima, Brazil. J. Med. Entomol., 2021; 58(6): 2488–2494. doi:10.1093/jme/tjab068. 33884431

[10] Wieczorek J, Bloom D, Guralnick R Darwin core: an evolving community-developed biodiversity data standard. PLoS ONE, 2012; 7(1): e29715. doi:10.1371/journal.pone.0029715. 22238640 PMC3253084

[11] GBIF: The Global Biodiversity Information Facility. What is GBIF? 2022; Available from https://www.gbif.org/what-is-gbif/. Accessed 27 Feb 2022.

[12] Andrade-Filho JD, Reis AL, Monteiro CC Fiocruz/COLFLEB - Coleção de Flebotomíneos. Version 1.50. FIOCRUZ - Oswaldo Cruz Foundation. 2022; Occurrence dataset 10.15468/sxcpfp.

[13] Shimabukuro PHF, de Andrade AJ, Galati EAB. Checklist of American sand flies (Diptera, Psychodidae, Phlebotominae): genera, species, and their distribution. Zookeys, 2017; (660): 67–106. doi:10.3897/zookeys.660.10508. PMC5549530 28794674

[14] Martins AV, Williams P, Falcão AL. American sand flies (Diptera: Psychodidae, Phlebotominae). Rio de Janeiro: Academia Brasileira de Ciências, 1978.

[15] Pinto Ide S, Chagas BD, Rodrigues AA DNA barcoding of neotropical sand flies (diptera, psychodidae, phlebotominae): species identification and discovery within Brazil. PLoS One, 2015; 10(10): e0140636. doi:10.1371/journal.pone.0140636. 26506007 PMC4624639

[16] FIOCRUZ.Coleções Biológicas. https://portal.fiocruz.br/colecoes-biologicas/. Accessed 27 February 2022.

[17] speciesLink. Centro de Referência em Informação Ambiental: Barão Geraldo. https://specieslink.net.

[18] Global Biodiversity Information Facility (GBIF) Information Processing Toolkit (IPT). Fiocruz/COLFLEB - Coleção de Flebotomíneos. http://ipt.fiocruz.br/ipt/resource?r=fiocruz_colfleb.

[19] de Souza Fernandes W, de Oliveira Moura Infran J, Falcão de Oliveira E Phlebotomine Sandfly (Diptera: Psychodidae) Fauna and The Association Between Climatic Variables and The Abundance of *Lutzomyia longipalpis* sensu lato in an Intense Transmission Area for Visceral Leishmaniasis in Central Western Brazil. J. Med. Entomol., 2022; tjac006. doi:10.1093/jme/tjac006. 35139201

[20] Estallo EL, Santana M, Martín ME Environmental effects on phlebotominae sand flies (Diptera:Phychodidae) and implications for sand fly vector disease transmission in Corrientes city, northern Argentina. An. Acad. Bras. Cienc., 2021; 93(suppl 3): e20191278. doi:10.1590/0001-3765202120191278. 34730738

[21] Vectors of human disease series. GigaByte. 2022; 10.46471/GIGABYTE_SERIES_0002.

