# Supplementary material for: Online catalogue of the Coleção de Flebotomíneos (FIOCRUZ/COLFLEB), a biological collection of American sand flies (Diptera: Psychodidae, Phlebotominae) held at Fiocruz Minas, Brazil
Source: GigaByte. 2022 May 23;2022:gigabyte52. doi: 10.46471/gigabyte.52 (PMC9930498; doi:10.46471/gigabyte.52)
Supplement: Translation

# Translation: Catálogo online da Coleção de Flebotomíneos (FIOCRUZ/COLFLEB), uma coleção biológica de flebotomíneos americanos (Diptera: Psychodidae, Phlebotominae) da Fiocruz Minas, Brasil

**Authors:** José Dilermando Andrade-Filho, Alanna Silva Reis, Carolina Cunha Monteiro, Paloma Helena Fernandes Shimabukuro

**Affiliations:** ^1^ Coleção de Flebotomíneos (FIOCRUZ/COLFLEB), Instituto René Rachou, Fiocruz Minas Avenida Augusto de Lima, 1715 - Barro Preto, 30190009, Belo Horizonte, Brazil; ^2^ Grupo de Estudos em Leishmanioses, Instituto René Rachou, Fiocruz Minas Avenida Augusto de Lima, 1715 - Barro Preto, 30190009, Belo Horizonte, Brazil

## Abstract

A Coleção de Flebotomíneos (“Coleção de Flebotomíneos”; FIOCRUZ/COLFLEB) da Fiocruz Minas é uma coleção biológica composta por aproximadamente 80.000 exemplares representando 370 espécies de flebotomíneos (Diptera: Psychodidae, Phlebotominae). Estes insetos foram coletados principalmente nas Américas nos últimos 80 anos por entomologistas interessados em entender e contribuir com conhecimento para o controle das leishmanioses, doenças causadas por protozoários transmitidos por vetores. Desde 2010, os metadados de cada espécime mantido na FIOCRUZ/COLFLEB, incluindo os dados retroativos daqueles depositados em décadas anteriores, estão sendo digitalizados. Aqui, apresentamos o catálogo eletrônico resultante contendo registros para 72.624 exemplares, incluindo todas as informações associadas disponíveis para cada espécime. O catálogo é publicado online por meio da rede speciesLink e do Sistema de Informação sobre a Biodiversidade Brasileira (SiBBr).

## Descrição dos dados

### Introdução

As coleções biológicas são repositórios de espécimes que abrigam a biodiversidade e suas informações relacionadas, que podem ser utilizados em diversas áreas de pesquisa científica [1]. Essas coleções são especialmente importantes para insetos de importância médica e veterinária, cuja identificação taxonômica precisa é necessária para entender e elaborar intervenções na epidemiologia de doenças de transmissão vetorial [2]. A “Coleção de Flebotomíneos” está localizada no Instituto René Rachou, Fiocruz Minas (FIOCRUZ/COLFLEB), instituição federal de pesquisa em saúde pública em Belo Horizonte, Minas Gerais, Brasil. FIOCRUZ/COLFLEB é uma coleção biológica de flebotomíneos (Diptera: Psychodidae, Phlebotominae) preservados em lâminas de microscopia. Esses pequenos insetos são de considerável importância médica e de saúde pública, pois as fêmeas adultas são hematófagas e transmitem parasitas protozoários do gênero *Leishmania*, agente etiológico das leishmanioses, além de outros patógenos bacterianos e virais, que infectam vertebrados humanos e não humanos [3]. Os espécimes de insetos depositados na FIOCRUZ/COLFLEB são provenientes de diversos projetos de pesquisa realizados ao longo dos últimos 80 anos. Esses projetos foram realizados em muitas áreas diferentes de transmissão de leishmaniose, bem como em ambientes silvestres onde a leishmaniose nunca foi registrada. Os flebotomíneos depositados geralmente foram coletados usando armadilhas de luz e foram identificados por especialistas altamente treinados usando chaves taxonômicas disponíveis [2, 4]. Os espécimes podem subsidiar pesquisas nas áreas de taxonomia e sistemática [5–7], e seus dados associados podem ser usados em modelagem de distribuição de nichos ecológicos/espécies [8], entre outras aplicações [9].

O conjunto de dados aqui apresentado inclui os metadados para cada espécime individual de flebotomíneo depositado na FIOCRUZ/COLFLEB desde 1953. Nosso conjunto de dados possui 57 campos para cada espécime individual de flebotomíneo descrevendo sua (i) taxonomia (kingdom, phylum, class, order, family, genus, specificEpithet, infraspecificEpithet, scientificName, scientificNameAuthorship, taxonRank, vernacularName, typestatus); (ii) informações de campo, incluindo os coletores (recordedBy), data de coleta, método de coleta, identificação da armadilha, descrição do sítio de coleta (occurrenceRemarks, eventDate, eventTime, habitat, samplingProtocol, samplingEffort, eventRemarks); (iii) dados geográficos (country, countryCode, stateProvince, county, Island, waterbody, locality, locationRemarks, decimalLongitude, decimalLatitude, georeferenceRemarks); e (iv) referências aos dados de tombamento e catalogação (occurrenceID, catalogNumber, OtherCatalogNumbers). Os dados associados a cada objeto físico de nossa coleção possuem um cartão de papel com diversos campos referentes ao depositante do espécime, localização, quantidade de espécimes etc. Os dados desses cartões foram digitalizados por um membro dedicado da equipe de coleta desde 2010. Os dados são fornecidos no formato Darwin Core [10]. Nossos dados estão disponíveis no Sistema de Informação sobre a Biodiversidade Brasileira (SiBBr), plataforma online que integra dados e informações sobre biodiversidade e ecossistemas. O SiBBr é o nó brasileiro do Global Biodiversity Information Facility (GBIF) [11], uma infraestrutura reconhecida internacionalmente para a disponibilização de dados da biodiversidade. Nosso conjunto de dados foi submetido ao GBIF e está disponível publicamente para uso por outros (veja a Figura 1) [12].

**Figura 1.** Mapa interativo das ocorrências georreferenciadas hospedadas pelo GBIF [12]. https://www.gbif.org/dataset/2a629a9a-38d1-496b-afbf-b4ff3b8fae60

### Contexto

Os flebotomíneos são insetos de importância médica, pois estão envolvidos na transmissão de patógenos entre animais humanos e não humanos. Aproximadamente 1.000 espécies de flebotomíneos foram descritas, das quais 530 são conhecidas por ocorrerem nas regiões neotropicais e neárticas [13].

A FIOCRUZ/COLFLEB foi iniciada oficialmente em 1953 como parte do trabalho dos pesquisadores Amilcar Vianna Martins e Alda Lima Falcão, em colaboração com o técnico João Evangelista da Silva [14]. O número de espécimes na coleção aumentou consideravelmente durante as décadas de 1960 e 1970. Nos últimos 20 anos, espécimes fósseis, bem como espécimes de comprovantes de estudos de código de barras baseados em DNA e outros estudos epidemiológicos envolvendo trabalho de campo, continuaram a ser depositados na coleção [7, 15].

A FIOCRUZ/COLFLEB contém 922 espécimes-tipo pertencentes a 151 espécies, incluindo holótipos, alótipos, parátipos, plesiótipos, cotipos, topótipos, homeótipos, síntipos e neótipos. A FIOCRUZ/COLFLEB também possui uma coleção diversificada de espécies fósseis de flebotomíneos neotropicais, atualmente composta por 47 âmbares da República Dominicana, dentro dos quais estão preservados 162 flebotomíneos de nove espécies. Além disso, a FIOCRUZ/COLFLEB possui mais de 700 espécimes-testemunhos depositados a partir de estudos de código de barras de DNA (DNA *barcoding*).

Esforços para digitalizar os metadados dos espécimes biológicos mantidos na FIOCRUZ/COFLEB estão em andamento desde 2010. Dentre as coleções biológicas das diversas instituições regionais que compõem a Fiocruz [16], a FIOCRUZ/COLFLEB tem maior número de registros online publicados. Nosso catálogo online também está integrado à rede speciesLink [17] e ao Sistema de Informação sobre a Biodiversidade Brasileira (SiBBr) [18].

## Métodos

Os flebotomíneos depositados na FIOCRUZ/COLFLEB são adultos e foram coletados por diversos métodos, incluindo coleta em repouso de superfícies artificiais e naturais com aspiradores mecânicos ou manuais (“pooters”); armadilhas de luz tipo CDC sem isca e com isca de CO_2_; e armadilhas Shannon. As coletas foram feitas em diferentes ambientes, incluindo doméstico e peridoméstico (ou seja, casas e seus arredores em áreas urbanas, suburbanas e rurais) e áreas silvestres e selvagens relativamente intocadas (por exemplo, áreas florestais remotas). Nossos exemplares são provenientes de todos os 27 estados do Brasil e de outros 19 países diferentes das Américas.

Os insetos capturados vivos são permanentemente montados em lâminas de microscópio de vidro e preservados em meio Berlese ou Bálsamo do Canadá, enquanto os espécimes fósseis são mantidos em frascos de plástico. Todos os tipos são rotulados com etiquetas codificadas por cores.

### Validação dos dados e controle de qualidade

Os insetos foram identificados por taxonomistas experientes usando chaves disponíveis na literatura: as de Galati [2] e Young e Duncan [4].

O conjunto de dados está no formato Darwin Core [10], com 57 termos disponíveis. Todos os campos obrigatórios estão presentes e passaram por triagem no IPT da FIOCRUZ (Integrated Publishing Toolkit) [18], que é o software do GBIF para fornecer dados através de sua rede. Os campos de metadados também estão disponíveis nas páginas online.

## Potencial de re-uso dos dados

Os dados associados aos espécimes biológicos depositados na FIOCRUZ/COLFLEB são importantes porque (i) descrevem a distribuição dos flebotomíneos em diferentes partes do Brasil, e (ii) têm uma boa cobertura temporal – alguns espécimes datam de 1939, enquanto o depósito mais recente foi registrado em 2021. Esses dados podem ser usados para muitas aplicações diferentes, em diferentes áreas de pesquisa, incluindo taxonomia e sistemática [5–7], e modelagem de nicho ecológico [8], entre outros [9], para subsidiar atividades de controle [19, 20].

## Data Availability

Os dados apresentados neste artigo são publicados através do IPT da FIOCRUZ – Fundação Oswaldo Cruz [18] e estão disponíveis sob licença CC0 no GBIF [12].

## Nota do editor

Este artigo faz parte de uma série de artigos de divulgação de dados disponibilizados pelo GBIF e apoiada pelo TDR, o Programa Especial de Pesquisa e Treinamento em Doenças Tropicais, da Organização Mundial da Saúde [21].

## Lista de abreviações

FIOCRUZ/COLFLEB: Coleção de Flebotomíneos, Fiocruz Minas; GBIF: Global Biodiversity Information Facility; IPT: Information Publishing Tool; SiBBr: Sistema de Informação sobre a Biodiversidade Brasileira; TDR: the Special Programme for Research and Training in Tropical Diseases.

## Aprovação ética

Não aplicável.

## Consentimento para publicação

Não aplicável.

## Financiamento

A FIOCRUZ/COLFLEB é mantida e financiada pela Fundação Oswaldo Cruz. JDAF recebeu bolsas de pesquisa do CNPq (302701/2016-8) e financiamento da Fundação de Amparo à Pesquisa do Estado de Minas Gerais (FAPEMIG; PPM-00792-18). PHFS recebeu bolsa de pesquisa da FAPEMIG (PPM-00676-18).

## Contribuições dos autores

JDAF: curador da coleção, fornecimento de recursos, revisão do manuscrito; ASR: curadoria de dados; CCM: curadoria de dados; PHFS: curadora da coleção, preparação do manuscrito.

## References

[1] KamenskiPA, SazonovAE, FedyaninAA Biological Collections: Chasing the Ideal. Acta Naturae, 2016; 8(2): 6–9.PMC494798427437135

[2] GalatiEAB. Classification of Phlebotominae. In: RangelEF, LainsonR (eds), Flebotomíneos do Brazil. Rio de Janeiro: Editora Fiocruz, 2003; pp. 23–175.

[3] RangelEF, LainsonR. Flebotomíneos do Brasil. In: RangelEF, LainsonR (eds), Flebotomíneos do Brazil. Rio de Janeiro: Editora Fiocruz, 2003.

[4] YoungDG, DuncanMA. Guide to the identification and geographic distribution of Lutzomyia sandflies in Mexico, The West Indies, Central and South America (Diptera: Psychodidae). Mem. Am. Entomol. Inst., 1994; 54: 1–881.

[5] CostaGDS, JúniorAMP, Arley Costa PessoaF New Records of Phlebotomine Sand Flies (Diptera: Psychodidae) From the Western Brazilian Amazon and the Description of the Female of Pintomyia fiocruzi. J. Med. Entomol., 2020; 57(4): 1328–1333. doi:10.1093/jme/tjaa030.32099998

[6] SábioPB, BrilhanteAF, QuintanaMG On the Synonyms of *Psathyromyia (Psathyromyia) shannoni* (Dyar, 1929) and *Pa. bigeniculata* (Floch & Abonnenc, 1941) and the Resuscitation of *Pa. pifanoi* (Ortiz, 1972) With the Description of Its Female (Diptera: Psychodidae: Phlebotominae). J. Med. Entomol., 2016; 53(5): 1140–1147. doi:10.1093/jme/tjw094.27358041

[7] RodriguesBL, BatonLA, ShimabukuroPHF. Single-locus DNA barcoding and species delimitation of the sandfly subgenus Evandromyia (Aldamyia). Med. Vet. Entomol., 2020; 34(4): 420–431. doi:10.1111/mve.12458.32590879

[8] Andrade-FilhoJD, ScholteRGC, AmaralALG Occurrence and Probability Maps of *Lutzomyia longipalpis* and *Lutzomyia cruzi* (Diptera: Psychodidae: Phlebotominae) in Brazil. J. Med. Entomol., 2017; 54(5): 1430–1434. doi:10.1093/jme/tjx094.28472338

[9] RodriguesBL, CostaGDS, ShimabukuroPHF. Identification of Bloodmeals from Sand Flies (Diptera: Psychodidae) Collected in the Parque Nacional do Viruá, State of Roraima, Brazil. J. Med. Entomol., 2021; 58(6): 2488–2494. doi:10.1093/jme/tjab068.33884431

[10] WieczorekJ, BloomD, GuralnickR Darwin core: an evolving community-developed biodiversity data standard. PLoS ONE, 2012; 7(1): e29715. doi:10.1371/journal.pone.0029715.22238640PMC3253084

[11] GBIF: The Global Biodiversity Information Facility. What is GBIF? 2022; Available from https://www.gbif.org/what-is-gbif/. Accessed 27 Feb 2022.

[12] Andrade-FilhoJD, ReisAL, MonteiroCC Fiocruz/COLFLEB - Coleção de Flebotomíneos. Version 1.50. FIOCRUZ - Oswaldo Cruz Foundation. 2022; Occurrence dataset 10.15468/sxcpfp.

[13] ShimabukuroPHF, de AndradeAJ, GalatiEAB. Checklist of American sand flies (Diptera, Psychodidae, Phlebotominae): genera, species, and their distribution. Zookeys, 2017; (660): 67–106. doi:10.3897/zookeys.660.10508.PMC554953028794674

[14] MartinsAV, WilliamsP, FalcãoAL. American sand flies (Diptera: Psychodidae, Phlebotominae). Rio de Janeiro: Academia Brasileira de Ciências, 1978.

[15] Pinto IdeS, ChagasBD, RodriguesAA DNA barcoding of neotropical sand flies (diptera, psychodidae, phlebotominae): species identification and discovery within Brazil. PLoS One, 2015; 10(10): e0140636. doi:10.1371/journal.pone.0140636.26506007PMC4624639

[16] FIOCRUZ.Coleções Biológicas. https://portal.fiocruz.br/colecoes-biologicas/. Accessed 27 February 2022.

[17] speciesLink. Centro de Referência em Informação Ambiental: Barão Geraldo. https://specieslink.net.

[18] Global Biodiversity Information Facility (GBIF) Information Processing Toolkit (IPT). Fiocruz/COLFLEB - Coleção de Flebotomíneos. http://ipt.fiocruz.br/ipt/resource?r=fiocruz_colfleb.

[19] de Souza FernandesW, de Oliveira Moura InfranJ, Falcão de OliveiraE Phlebotomine Sandfly (Diptera: Psychodidae) Fauna and The Association Between Climatic Variables and The Abundance of *Lutzomyia longipalpis* sensu lato in an Intense Transmission Area for Visceral Leishmaniasis in Central Western Brazil. J. Med. Entomol., 2022; tjac006. doi:10.1093/jme/tjac006.35139201

[20] EstalloEL, SantanaM, MartínME Environmental effects on phlebotominae sand flies (Diptera:Phychodidae) and implications for sand fly vector disease transmission in Corrientes city, northern Argentina. An. Acad. Bras. Cienc., 2021; 93(suppl 3): e20191278. doi:10.1590/0001-3765202120191278.34730738

[21] Vectors of human disease series. GigaByte. 2022; 10.46471/GIGABYTE_SERIES_0002.
